# Cell-free DNA 5-hydroxymethylcytosine profiles of long non-coding RNA genes enable early detection and progression monitoring of human cancers

**DOI:** 10.1186/s13148-021-01183-6

**Published:** 2021-10-24

**Authors:** Meng Zhou, Ping Hou, Congcong Yan, Lu Chen, Ke Li, Yiran Wang, Jingting Zhao, Jianzhong Su, Jie Sun

**Affiliations:** School of Biomedical Engineering, School of Ophthalmology & Optometry and Eye Hospital, Wenzhou Medical University, Wenzhou, 325027 China

**Keywords:** Cell-free DNA, 5-hydroxymethylcytosine, Long non-coding RNAs

## Abstract

**Background:**

5-Hydroxymethylcytosine (5hmC) is a significant DNA epigenetic modification. However, the 5hmC modification alterations in genomic regions encoding long non-coding RNA (lncRNA) and their clinical significance remain poorly characterized.

**Results:**

A three-phase discovery–modeling–validation study was conducted to explore the potential of the plasma-derived 5hmC modification level in genomic regions encoding lncRNAs as a superior alternative biomarker for cancer diagnosis and surveillance. Genome-wide 5hmC profiles in the plasma circulating cell-free DNA of 1632 cancer and 1379 non-cancerous control samples from different cancer types and multiple centers were repurposed and characterized. A large number of altered 5hmC modifications were distributed at genomic regions encoding lncRNAs in cancerous compared with healthy subjects. Furthermore, most 5hmC-modified lncRNA genes were cancer-specific, with only a relatively small number of 5hmC-modified lncRNA genes shared by various cancer types. A 5hmC-LncRNA diagnostic score (5hLD-score) comprising 39 tissue-shared 5hmC-modified lncRNA gene markers was developed using elastic net regularization. The 5hLD-score was able to accurately distinguish tumors from healthy controls with an area under the curve (AUC) of 0.963 [95% confidence interval (CI) 0.940–0.985] and 0.912 (95% CI 0.837–0.987) in the training and internal validation cohorts, respectively. Results from three independent validations confirmed the robustness and stability of the 5hLD-score with an AUC of 0.851 (95% CI 0.786–0.916) in Zhang’s non-small cell lung cancer cohort, AUC of 0.887 (95% CI 0.852–0.922) in Tian’s esophageal cancer cohort, and AUC of 0.768 (95% CI 0.746–0.790) in Cai’s hepatocellular carcinoma cohort. In addition, a significant association was identified between the 5hLD-score and the progression from hepatitis to liver cancer. Finally, lncRNA genes modified by tissue-specific 5hmC alteration were again found to be capable of identifying the origin and location of tumors.

**Conclusion:**

The present study will contribute to the ongoing effort to understand the transcriptional programs of lncRNA genes, as well as facilitate the development of novel invasive genomic tools for early cancer detection and surveillance.

**Supplementary Information:**

The online version contains supplementary material available at 10.1186/s13148-021-01183-6.

## Background

Circulating cell-free DNA (cfDNA) is degraded DNA fragments released into the blood plasma as a result of cell death in different tissues. cfDNA has important properties and can be used to provide a proof-of-principle approach for the screening, early detection and monitoring of human cancer [1]. Liquid biopsy, a well-known minimally invasive technique, has notable advantages over existing diagnostic and prognostic methods and has attracted considerable public attention as a diagnostic method for cancer [2].

Epigenetic DNA modifications play diverse biological functions by modulating gene expression at the level of chromatin structure and organization [3]. Developments in chemical and biological technologies have led to the identification of a number of types of chemical modifications in DNA [4]. 5-Hydroxymethylcytosine (5hmC) is a novel and major epigenetic DNA modification resulting from 5-methylcytosine (5mC) oxidation by the ten-eleven translocation proteins [5]. Increasing evidence shows that 5hmC is not only the intermediate product of 5mC demethylation but also a stable epigenetic marker [6]. Highly variable global 5hmC content exists in normal human tissues and is not correlated with global 5mC content [7]. Previous genome-wide 5hmC profiling studies have demonstrated that 5hmC modification was preferentially distributed in tissue-specific enhancers, gene bodies and promoters, and that the gene-level 5hmC modifications were associated with gene expression status [8, 9]. 5hmC modification plays a crucial role in a wide range of physiological and pathological processes, and its aberrant alterations have been identified as a hallmark of carcinogenesis [10–12]. A series of recent studies have paid more attention to the identification of 5hmC signaling changes in cfDNA to characterize various potential patient conditions using highly sensitive and robust 5hmC sequencing technologies, such as Nano-hmC-Seal and hMe-Seal [2, 13], which highlighted 5hmC in cfDNA as a highly sensitive and reliable minimally invasive marker for cancer diagnosis and prognosis [2, 13–16].

Long non-coding RNAs (lncRNAs) are well-known important regulators of gene expression at the transcriptional and post-transcriptional levels through various mechanisms [17, 18]. Studies have indicated that lncRNAs are involved in nearly all key biological processes, and their aberrant expression has been widely reported in various types of cancer [19–21]. Recent studies have indicated that lncRNA gene expression could also be regulated by epigenetic DNA modifications, such as DNA methylation and histone modifications [22–24]. Despite extensive epigenetic DNA modification studies focusing on 5hmC modification, the majority of them only examined 5hmC modification distributed at protein-coding gene bodies and promoters and neglected the potential 5hmC alterations in genomic regions encoding lncRNAs and their clinical significance.

**Fig. 1:**
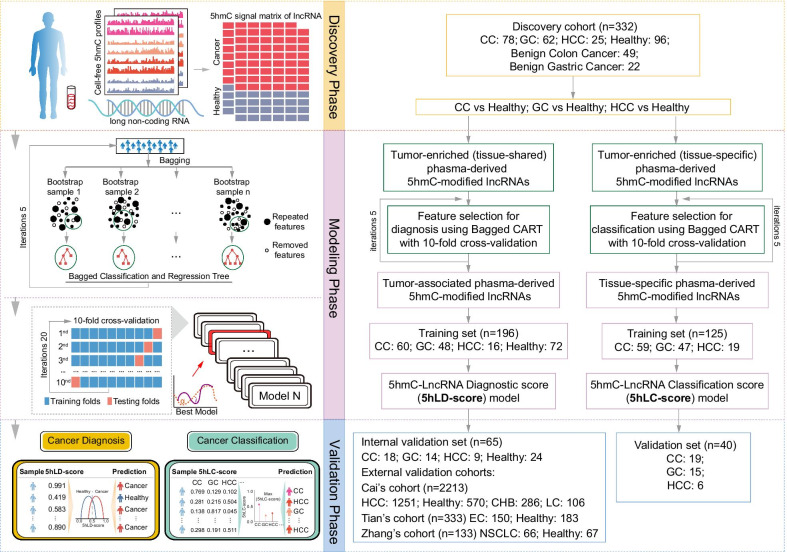
Workflow diagram of the study design. A three-phase discovery–modeling–validation study was conducted, including a total of 3011 samples (1632 cancers and 1379 non-cancerous samples). During the discovery phase, plasma-derived abnormal 5hmC-modified lncRNA genes were identified in Li’s cohort comprising HCC (*n* = 25), CC (*n* = 78), GC (*n* = 62) and healthy (*n* = 96) samples. The 5hmC-modified lncRNA-based predictive models for cancer diagnosis and classification were then constructed using Bagged CART with tenfold cross-validation and elastic net regularization in the training cohort, followed by validation in different independent cohorts from multiple centers. lncRNA, long non-coding RNA; CART, classification and regression tree; CC, colon cancer; CHB, chronic hepatitis B virus infection; EC, esophageal cancer; GC, gastric cancer; HCC, hepatocellular carcinoma; NSCLC, non-small-cell lung cancer; 5hmC, 5-hydroxymethylcytosine; 5hLD-score, 5hmC-LncRNA diagnostic score; 5hLC-score, 5hmC-lncRNA classification score

In the present study, genome-wide 5hmC profiles in plasma cfDNA from 1379 non-cancerous controls and 1632 cancer samples from different cancer types and multiple centers were repurposed and integrated to explore 5hmC alterations in genomic regions encoding lncRNAs in cancer. Next, a three-phase discovery–modeling–validation study was conducted to explore the potential of plasma-derived 5hmC modification level in genomic regions encoding lncRNAs as an alternative, superior biomarker for cancer diagnosis and surveillance (Fig. 1).

## Results

### Plasma-derived lncRNA genes with abnormal 5hmC modifications in malignant tumors

**Fig. 2:**
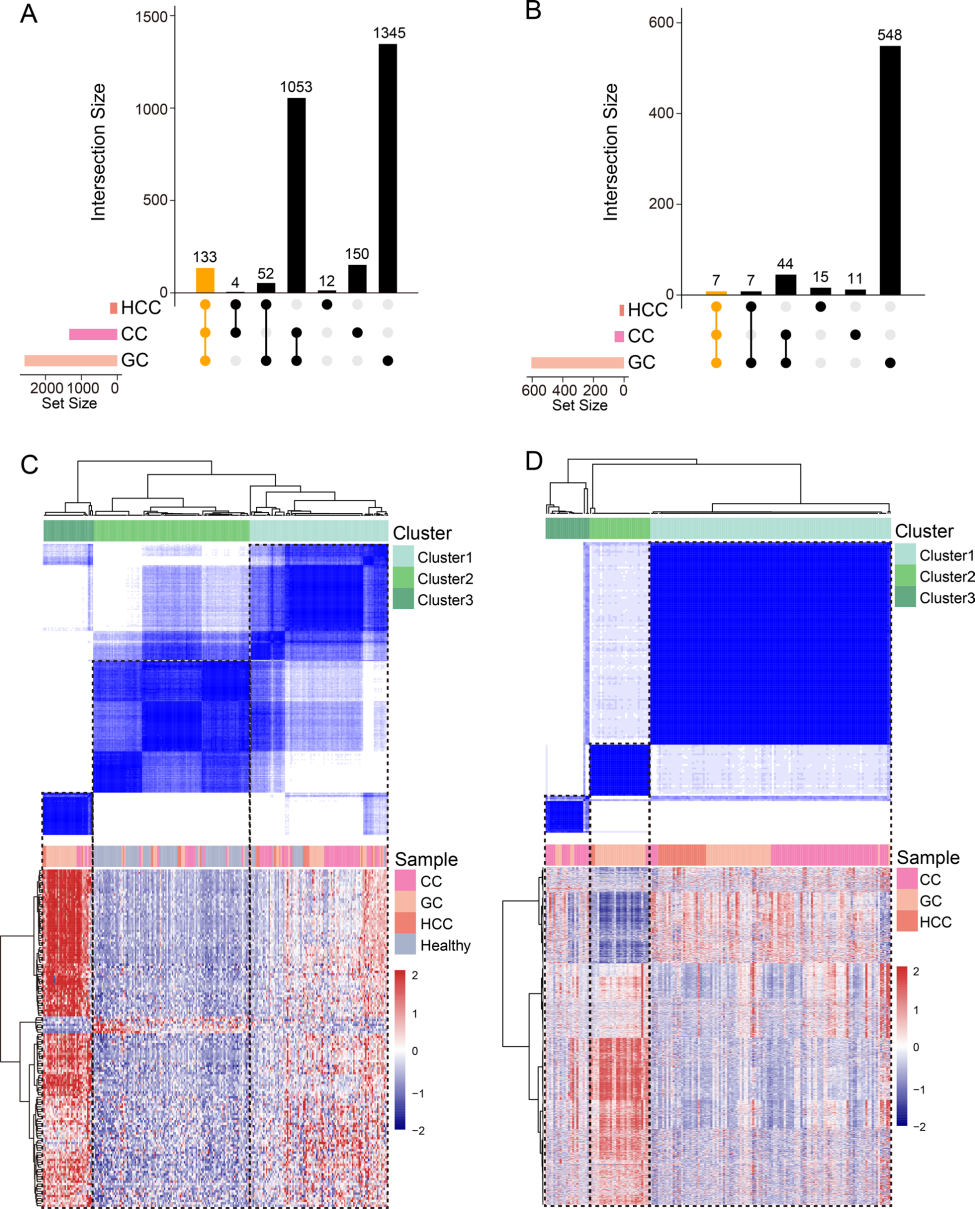
Tissue relevance of tumor-enriched 5hmC-modified lncRNA genes. UpSet plots visualizing the intersections of 5hmC-modified **A** positively and **B** negatively enriched lncRNAs in different cancer types compared with healthy controls. Consensus clustering and heatmap of **C** tissue-shared and **D** tissue-specific 5hmC-modified lncRNA genes. lncRNA, long non-coding RNA; CC, colon cancer; GC, gastric cancer; HCC, hepatocellular carcinoma

To examine the impact of 5hmC in genomic regions encoding lncRNAs in various cancer types, the 5hmC profiles of lncRNA genes were compared among hepatocellular carcinoma (HCC, *n* = 25), colon cancer (CC, *n* = 78), gastric cancer (GC, *n* = 62) and healthy (*n* = 96) samples from Li’s cohort. Finally, a total of 1402 lncRNA genes (1340 with significantly increased and 62 with significantly decreased 5hmC levels), 3189 (2583 with significantly increased and 606 with significantly decreased 5hmC levels) and 230 (201 with significantly increased and 29 with significantly decreased 5hmC levels) were identified as tumor-related 5hmC-modified lncRNA genes in CC, GC and HCC compared with healthy samples, respectively (Fig. 2A, B). Specifically, 140 tumor-related 5hmC-modified lncRNA genes were shared by three cancer types and 2081 were found to have tissue-specific differential 5hmC modifications (Additional file 1: Table S1). To further verify the relationship between tissue-shared lncRNA genes and samples, consensus clustering was performed on 140 tissue-shared 5hmC-modified lncRNA genes based on the 5hmC profiles in genomic regions encoding lncRNAs, which identified three distinct sample clusters; clusters 1 and 3 mainly exhibited tumor samples and cluster 2 healthy samples (Fig. 2C). Similarly, consensus clustering of 2081 tissue-specific 5hmC-modified lncRNA genes revealed three distinct patient clusters, with different tumor types clearly distinguishable among them (Fig. 2D). These results suggested that tumor-related plasma-derived 5hmC-modified lncRNA gene profiles differ significantly depending on the tissue source and can be used to guide liquid biopsy in patients.

### Establishment of a diagnostic model for the early detection of tumors based on tumor-shared 5hmC-modified lncRNA genes

**Fig. 3:**
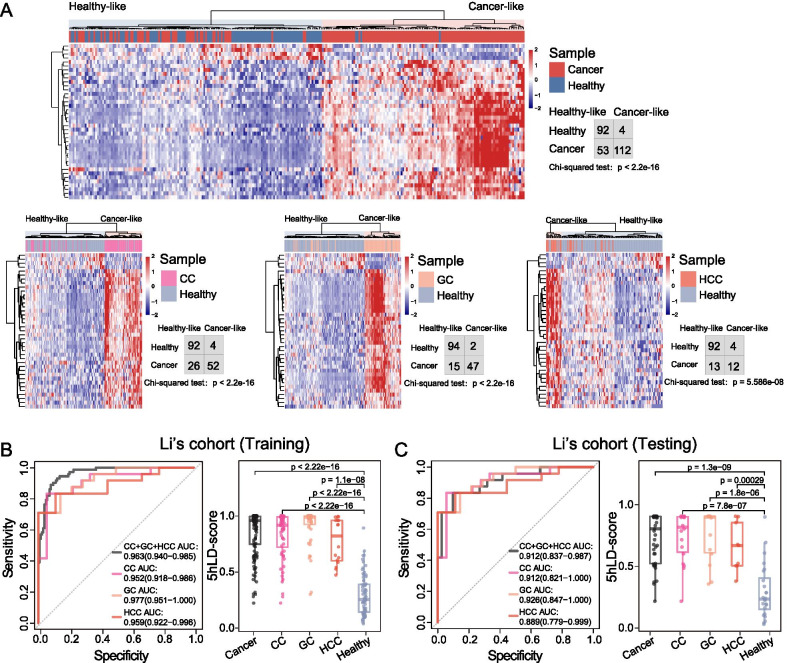
Development and internal validation of a diagnostic model (5hLD-score) based on tumor-shared 5hmC-modified lncRNA genes. **A** Unsupervised hierarchical clustering and heatmap of tissue-shared 5hmC-modified lncRNA gene markers in the training cohort. Statistical significance was determined using Chi-square test. Receiver operating characteristic curve (left panel) and boxplot (right panel) of the 5hLD-score for cancer detection in the **B** training and **C** internal validation cohorts. Statistical significance was determined using Wilcoxon rank-sum test. CC, colon cancer; GC, gastric cancer; HCC, hepatocellular carcinoma; AUC, area under the curve; 5hLD-score, 5hmC-LncRNA diagnostic score

To identify 5hmC-modified lncRNA genes that could be used as diagnostic biomarkers, feature selection was performed on 140 tissue-shared 5hmC-modified lncRNA genes using RFE based on CART, and 39 were identified as optimal diagnostic markers (Additional file 2: Table S2). Unsupervised hierarchical clustering revealed a clear separation between tumors and non-tumors (*χ*^2^ *P* < 2.2e−16 for CC and GC, and *P* = 5.586e−08 for HCC; Fig. 3A).

Next, a discovery–validation experiment was conducted by splitting samples from Li’s cohort into the training and internal validation cohort, using other, completely independent, cohorts for external validation. The workflow for the construction and validation of the 5hLD-score is shown in Fig. 1. Using the elastic net in the discovery cohort, the 5hLD-score was established, comprising 39 tissue-shared 5hmC-modified lncRNA gene markers that effectively distinguished tumors from non-tumors with an overall AUC of 0.963 [95% confidence interval (CI) = 0.940–0.985; Fig. 3B). For different cancer types, the 5hLD-score had an AUC value of 0.952 (95% CI 0.918–0.986) for CC detection, 0.977 (95% CI 0.951–1.000) for GC detection and 0.959 (95% CI 0.922–0.996) for HCC detection, respectively (Fig. 3B). When tested in the internal validation cohort, the 5hLD-score differentiated tumors from healthy controls with an overall AUC of 0.912 (95% CI 0.837–0.987), 0.912 (95% CI 0.821–1.000) for CC detection, 0.926 (95% CI 0.847–1.000) for GC detection and 0.889 (95% CI 0.779–0.999) for HCC detection, respectively (Fig. 3C). In addition, the 5hLD-score derived from tumors was significantly higher than that of healthy controls in the training and internal validation cohorts (Fig. 3B, C). These results highlighted the diagnostic performance of the 5hLD-score for cancer detection.

### Independent validation of the 5hLD-score

**Fig. 4:**
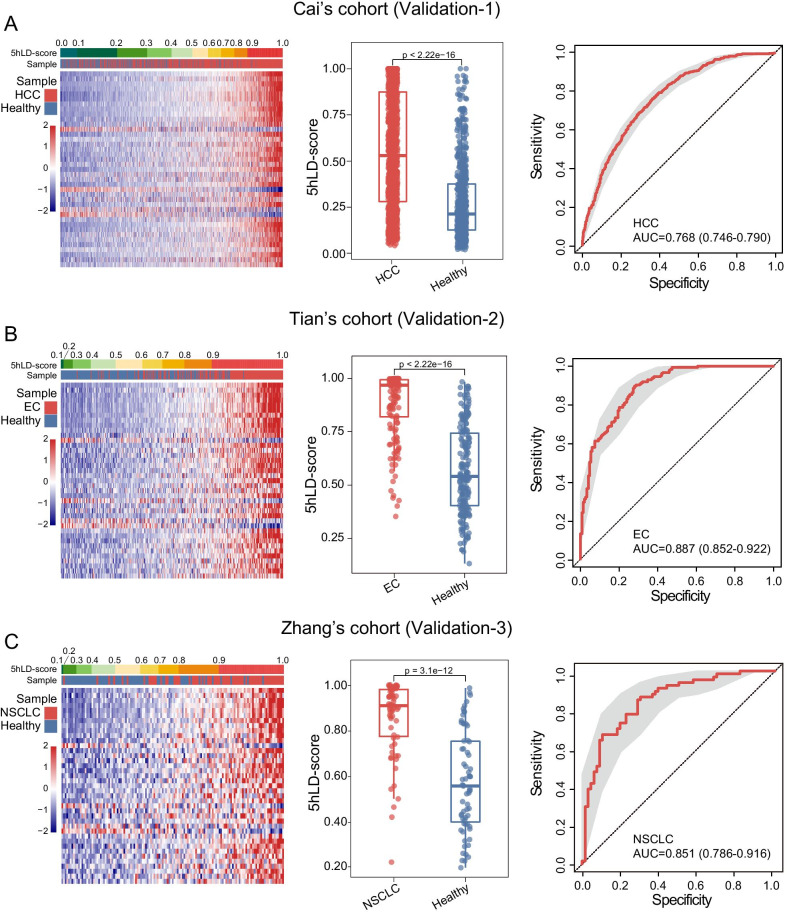
Further independent validation of the 5hLD-score. The 5hmC modification heatmap, boxplots and receiver operating characteristic curve of the 5hLD-score in the **A** validation-1 cohort comprising 1251 HCC and 570 healthy control samples, **B** validation-2 cohort comprising 150 EC and 183 healthy control samples and **C** validation-3 cohort comprising 66 NSCLC and 67 healthy control samples. Statistical significance was determined using the Wilcoxon rank-sum test. HCC, hepatocellular carcinoma; EC, esophageal cancer; NSCLC, non-small-cell lung cancer; AUC, area under the curve; 5hLD-score, 5hmC-LncRNA diagnostic score

To evaluate the reliability and robustness of the 5hLD-score, the predictive power of the 5hLD-score was further tested in three completely independent cohorts from multiple centers. In Cai’s cohort of 1251 HCC and 570 healthy controls, HCC samples exhibited a higher 5hLD-score than healthy controls (Wilcoxon rank-sum test *P* < 2.22e−16; Fig. 4A). The 5hLD-score achieved a high predictive power for distinguishing HCC from healthy controls with an AUC of 0.768 (95% CI 0.746–0.790; Fig. 4A). Another independent cohort (Tian’s cohort), including 150 esophageal cancer (EC) and 183 healthy samples, was different from cancer types of the training cohort. As shown in Fig. 4B, it was found that, the higher the 5hLD-score of a sample, the higher the likelihood of a sample being cancerous (Fig. 4B). Similarly, the 5hLD-score of EC samples was significantly higher than that of healthy samples (Wilcoxon rank-sum test *P* < 2.22e−16; Fig. 4B). The 5hLD-score was again found to effectively distinguish EC from healthy controls, with an AUC of 0.887 (95% CI 0.852–0.922; Fig. 4B). Finally, the 5hLD-score model was further evaluated in Zhang’s cohort of 66 non-small-cell lung cancer (NSCLC) and 67 healthy samples that were different from the training cohort. Results from Zhang’s cohort indicated that the 5hLD-score was significantly higher in NSCLC samples compared with healthy controls (Wilcoxon rank-sum test; *P* = 3.1e−12) and achieved an AUC of 0.851 (95% CI 0.786–0.916) in distinguishing NSCLC samples from healthy controls (Fig. 4C). These results confirmed the robustness and stability of the 5hLD-score in distinguishing cancerous from healthy samples.

### Association between the 5hLD-score and disease progression

**Fig. 5:**
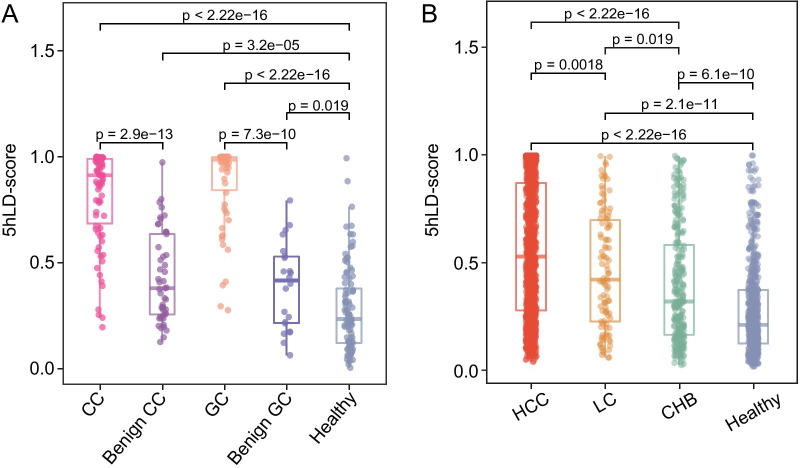
Association between the 5hLD-score and disease progression. **A** Boxplots showing the distribution of the 5hLD-score in cancer patients and patients with benign diseases. **B** Boxplots showing the association between the 5hLD-score and HCC progression. Statistical significance was determined using the Wilcoxon rank-sum test. CC, colon cancer; CHB, chronic hepatitis B virus infection; GC, gastric cancer; HCC, hepatocellular carcinoma; 5hLD-score, 5hmC-LncRNA diagnostic score; LC, liver cirrhosis

It was further examined whether the 5hLD-score can be used to monitor disease progression. First, the 5hLD-scores were compared between tumor, benign tumor and healthy control samples from Li’s cohort, and it was found that the 5hLD-score of tumor samples was significantly higher than that of benign tumor samples (Wilcoxon rank-sum test, *P* = 2.9e−13 for CC vs. benign colon and *P* = 7.3e−10 for GC vs. benign gastric), whereas benign tumors also exhibited a significantly higher 5hLD-score compared with healthy controls (Wilcoxon rank-sum test, *P* = 3.2e−05 for benign colon and *P* = 0.019 for benign gastric; Fig. 5A). In addition, the 5hLD-score was used in HCC, liver cirrhosis (LC) and chronic hepatitis B virus infection (CHB) samples from an independent cohort (Cai’s cohort), and found that samples with different liver diseases exhibited a significantly higher 5hLD-score than healthy controls (Wilcoxon rank-sum test, *P* < 2.22e−16 for HCC, *P* = 2.1e−11 for LC and *P* = 6.1e−10 for CHB; Fig. 5B). Furthermore, during the progression from hepatitis to liver cancer, the patient’s 5hLD-score exhibited a clear increasing trend from CHB to HCC (Fig. 5B). As shown in Fig. 5B, HCC samples had a significantly higher 5hLD-score compared with LC (Wilcoxon rank-sum test, *P* = 0.0018) and CHB (Wilcoxon rank-sum test, *P* < 2.22e−16), whereas the 5hLD-score in LC samples was significantly higher than that in CHB samples (Wilcoxon rank-sum test, *P* = 0.019; Fig. 5B). These findings indicated that the 5hLD-score was associated with disease progression, and might be useful for monitoring disease progression.

### Potential of tissue-specific plasma-derived 5hmC-modified lncRNA genes as a noninvasive biomarker for identifying tumor origin

**Fig. 6:**
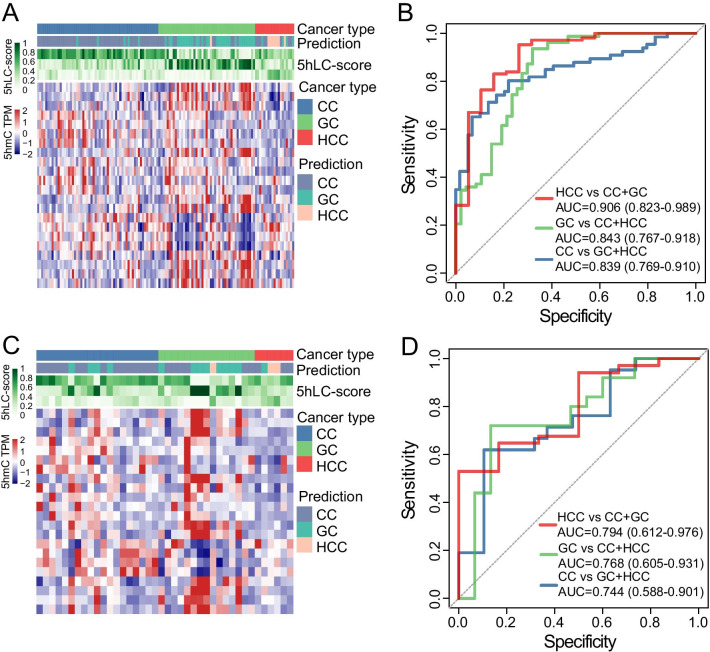
Development and internal validation of a classification model (5hmC-score) based on tissue-specific plasma-derived 5hmC-modified lncRNA genes. Heatmap showing the distribution of the 5hmC-score and 5hmC levels for different cancer types in the **A** training and **C** independent validation cohorts. ROC curve and AUC value of the 5hmC-score for identifying tumor origin and location in the **B** training and **D** independent validation cohorts. AUC, area under the ROC curve; CC, colon cancer; GC, gastric cancer; HCC, hepatocellular carcinoma; 5hLC-score, 5hmC-lncRNA classification score; TPM, transcripts per million

To investigate the potential of 5hmC alterations in genomic regions encoding lncRNAs as a noninvasive biomarker for identifying tumor origin, feature selection was performed on 2081 tumor-related tissue-specific 5hmC-modified lncRNA genes, and the 5hLC-score comprising 22 optimal 5hmC-modified lncRNA genes was established to identify cancer types in the training cohort. As shown in Fig. 6A, the 5hmC profiles in genomic regions encoding lncRNAs were different among patients with different cancer types, and the 5hLC-score was very accurate in predicting cancer types, with an AUC of 0.906 (95% CI 0.823–0.989) in distinguishing HCC, AUC of 0.843 (95% CI 0.767–0.918) in distinguishing GC and AUC of 0.839 (95% CI 0.769–0.910) in distinguishing CC (Fig. 6B), from other cancer types. Following testing in an independent validation cohort, the 5hLC-score was again shown capable of distinguishing between patients with different cancer types, with an AUC of 0.794 (95% CI 0.612–0.976) for HCC, 0.768 (95% CI 0.605–0.931) for GC and 0.744 (95% CI 0.588–0.901) for CC (Fig. 6C, D). These results demonstrated that the 5hLC-score could be used to identify the origin and location of tumors.

## Discussion

Liquid biopsy is a highly effective method of early cancer detection and tumor classification [25, 26]. Recent studies have used the novel Nano-hmC-Seal technology to generate the vast genome-wide profiles of 5hmC in cfDNA from blood plasma for various cancer types [2]. A growing body of evidence from 5hmC profile analysis in cfDNA has indicated that 5hmC signal changes could serve as valuable noninvasive biomarkers to improve the sensitivity, specificity and accuracy of existing clinical methods for cancer diagnosis, prognosis and surveillance. However, the majority of previous studies about the association between 5hmC and gene expression have focused on 5hmC modification patterns in protein-coding gene bodies and promoters in cfDNA from blood plasma. The 5hmC modification alterations in genomic regions encoding lncRNAs and their clinical significance remain poorly characterized.

Recently, lncRNA has been confirmed to be involved in the regulation of important biological functions that determine cell fate and influenced a series of physiological and pathological states [27]. lncRNA promoters were found to have different epigenetic alteration patterns in cancer compared with protein-coding genes [28]. Hu et al*.* reported that tissue-derived 5hmC played a crucial role in regulating the transcription of lncRNA and served as a novel biomarker for prognosis in colorectal cancer. However, whether plasma-derived 5hmC-modified lncRNA genes is a critical biomarker for diagnosing cancer and distinguishing the type of cancer remains unclear. Therefore, the present study explored the potential of the plasma-derived 5hmC modification level in genomic regions encoding lncRNAs being used as an alternative, superior biomarker for cancer diagnosis and monitoring.

Herein, by repurposing 5hmC sequencing reads to genomic regions encoding lncRNAs, 5hmC alterations in genomic regions encoding lncRNAs were characterized in multiple cancer types, including HCC, CC and GC. A large number of altered 5hmC modifications were found to be distributed at lncRNA genes in patients with cancer compared with healthy subjects. Furthermore, only a relatively small number of 5hmC-modified lncRNA genes were common among different types of cancers, with the majority being cancer-specific. Using these tissue-shared 5hmC-modified lncRNA genes, feature selection was performed and a 5hLD-score was developed, which distinguished tumors from healthy controls with a high diagnostic performance in the training and internal validation cohorts. Indeed, some of these tissue-shared 5hmC-modified lncRNA genes in the 5hLD-score have been reported to play critical roles in cancer initiation, metastasis and prognosis. For instance, membrane-associated guanylate kinase inverted 1 intronic transcript has been well established to control cell proliferation in several cancer types [27, 29]. Recent in vitro and in vivo studies have demonstrated that SOX9 antisense RNA 1 and long intergenic non-protein-coding RNA 1124 (*LINC01124*) regulated HCC progression and metastasis by acting as competitive endogenous RNAs [30, 31]. Several other lncRNAs, such as *SERTAD4-antisense 1*, *LINC01124*, *AC011294.1* and RBPMS Antisense RNA 1, have also been associated with cancer initiation and prognosis [32–35].

Although the tissue-shared 5hmC-modified lncRNA genes and the 5hLD-score identified and developed were limited to three cancer types (HCC, GC and CC), the 5hLD-score also had a high and stable diagnostic performance for other cancer types, such as EC and NSCLC. The adoption of independent validation cohorts from different cancer types and multiple centers demonstrated that the superior diagnostic performance of the 5hLD-score observed in different patient cohorts was not due to the overfitting of data or cancer types, but that it is a stable and robust predictive score that could be extended to other types of cancer. Furthermore, unlike previous linear scoring models in which risk score thresholds needed to be trained, the 5hLD-score was designed with a range of 0–1.0, representing the final probability of tumors in each sample, markedly improving its potential for clinical application. In addition, a significant association was observed between the 5hLD-score and the progression from hepatitis to liver cancer, suggesting a promising potential of the 5hLD-score as a highly effective individualized guide for the monitoring and surveillance of disease progression.

Although, to the best of our knowledge, the 5hLD-score is the first diagnostic genomic tool based on 5hmC alterations in genomic regions encoding lncRNAs, the present study was not without its limitations. First, major clinical variables, such as follow-up time, were not controlled in this study; further independent validation studies combining clinical variables will help address problems such as the potential selection bias for model construction or clarify the implications of potential confounding variables. Secondly, the regulatory mechanism of 5hmC and these lncRNA genes remains unclear due to the unavailability of lncRNA expression profiles. Therefore, further functional studies on 5hmC-modified lncRNA genes are required to elucidate how 5hmC regulated lncRNA transcription is involved in oncogenesis and whether tumor type-specific 5hmC enrichment at non-coding regions in cfDNA is positively associated with non-coding RNA expression in tumor tissues. Finally, although the 5hLD-score has been validated in two other independent cancer cohorts, further validation in other retrospective or prospective cohorts is required to demonstrate the generalizability of the 5hLD-score for various cancer types.

## Conclusions

Collectively, the present study systemically characterized the alteration patterns of 5hmC modifications in genomic regions encoding lncRNAs in cancer, contributing to the ongoing effort in understanding the transcriptional programs of lncRNAs. Furthermore, the clinical relevance of 5hmC modifications in genomic regions encoding lncRNAs was investigated, and a clinically useful 5hmC-modified lncRNA-based scoring model that will pave the way for developing novel invasive genomic tools for early detection cancer detection and surveillance was developed and validated.

## Methods

### Samples and 5hmC epigenetic datasets

Genome-wide 5hmC profiles from plasma cfDNA samples were collected from previously published studies. A total of 3011 samples (1632 cancer and 1379 non-cancerous) were included in the present study: 78 colon cancer (CC), 62 gastric cancer (GC), 49 benign colon, 22 benign gastric and 25 hepatocellular carcinoma (HCC) samples, and 96 samples from healthy individuals from Li’s study (the NCBI Sequence Read Archive SRP080977) (hereinafter referred to as Li’s cohort) [2]; 1251 HCC, 106 liver cirrhosis (LC) and 286 chronic hepatitis B virus infection (CHB) samples and 570 healthy samples from Cai’s study (the NCBI Sequence Read Archive SRP137706) (hereinafter referred to as Cai’s cohort) [36]; 150 esophageal cancer (EC) samples and 183 samples from healthy individuals from Tian’s study (Genome Sequence Archive CRA000617) (hereinafter referred to as Tian’s cohort) [15]; 66 non-small-cell lung cancer (NSCLC) samples and 67 samples from healthy individuals from Zhang’s study (Genome Sequence Archive CRA000872) (hereinafter referred to as Zhang’s cohort) [16]. Detailed information on the samples and 5hmC epigenetic datasets used in this study is summarized in Table 1.

**Table 1:** Detailed information of plasma cfDNA samples across different cancer types used in this study

Cohorts	Sample status	Sample number	Data source
Li’s cohort		332	SRP080977
	Gastric cancer(GC)	62	
	Benign gastric cancer	22	
	Colon cancer(CC)	78	
	Benign colon cancer	49	
	Hepatocellular carcinoma (HCC)	25	
	Healthy	96	
Cai’s cohort		2213	SRP137706
	Chronic hepatitis B virus infection (CHB)	286	
	Hepatocellular carcinoma (HCC)	1251	
	Liver cirrhosis	106	
	Healthy	570	
Tian’s cohort		333	CRA000617
	Esophageal cancer (EC)	150	
	Healthy	183	
Zhang’s cohort		133	CRA000872
	Non-small-cell lung cancer (NSCLC)	66	
	Healthy	67	

### Data preprocessing and genome-wide mapping of 5hmC-modified lncRNAs

5hmC sequencing reads were aligned to the human genome GRCh37 using Bowtie2 (version 2.3.4.2) [37] with default parameters. The samples with extremely abnormal mapping rates were removed. SAM files were converted into BAM files and sorted using SAMtools (version 1.9) [38]. Unique non-duplicate matches to the genome were retained using Picard v.2.18.4 (http://broadinstitute.github.io/picard/). The released version of the lncRNA reference gene annotation file (GRCh38 version 34) was downloaded from the GENCODE database (https://www.gencodegenes.org/). LiftOver was used to transfer the mapping information from the GRCh38 version of the lncRNA reference gene annotation file to the GRCh37 version. Genes encoding lncRNAs were extracted based on GRCh37 annotation. Read counts of 5hmC-modified lncRNAs were calculated using the fragment counts in each RefSeq lncRNA obtained by BEDtools (version 2.27.1) [39]. The read counts were converted into Transcripts Per Kilobase of 5hmC in lncRNA per million mapped reads. Finally, 5hmC profiles of 16,827 lncRNAs were obtained for further analysis.

### Identification of 5hmC-modified lncRNA markers

To identify putative 5hmC-modified lncRNA gene markers, the 5hmC profiles of lncRNAs were first compared among CC, GC, HCC and healthy samples. The lncRNAs with differential 5hmC profiles were identified using the DESeq2 package (version 1.22.2) [40]. lncRNAs with a |log2foldchange| > 0.58 and false discovery rate adjusted *P* < 0.05 were selected as tumor-related 5hmC-modified lncRNAs. The recursive feature elimination (RFE) based on the Bagged classification and regression tree (CART) was used on these differentially 5hmC-modified lncRNAs to determine the optimal 5hmC-modified lncRNA markers with the best accuracy in distinguishing cancer from non-cancerous samples or between different cancer types, respectively. The marker selection process was conducted using out-of-fold performance on five repetitions of tenfold cross-validation analysis of the training cohort, and the model leading to the maximum “Accuracy” was selected. The feature selection was conducted using the “rfe” and “treebagFuncs” functions from the Caret (version 6.0-86) R package.

### Development of clinically predictive models for cancer diagnosis and classification

The elastic net regularization on a multivariable logistic regression model was used to develop a clinically predictive model capable of distinguishing between cancer and non-cancerous samples or between cancer types. The model was trained with tenfold cross-validation and optimized using a receiver operating characteristic (ROC) curve for a grid of parameter values for *α* and *λ* (*α* range, 0.05 to 1.00 with a length = 10; *λ* range: from 10^–1^ to 5 × 10^–1^ with a 0.1 increment), where *α* controls the relative proportion between the Ridge and Lasso penalty, and *λ* the overall strength of the penalty. This selection process was repeated 20 times. Finally, a diagnostic score model was obtained based on tumor-shared 5hmC-modified lncRNA markers [termed the 5hmC-LncRNA diagnostic score (5hLD-score) 5hLD-score]. The 5hLD-score range was 0–1.0 and represented a final probability of tumors for each sample. A classification score model based on tumor-specific 5hmC-modified lncRNA markers (termed the 5hLC-score) was developed to produce a risk score (range, 0–1.0) for each cancer type for each sample. (Each sample was assigned a specific cancer type with the highest risk score.)

### Statistical analysis

Statistical significance was performed for continuous values using Wilcoxon rank-sum test for two-group comparisons, Kruskal–Wallis test for multiple-group comparisons and Chi-square test for categorical variables unless otherwise specified in the figure legend. Consensus clustering was performed using the R package ‘ConsensusClusterPlus’ (version 1.48.0) [41], which can automatically select the number of clusters and is the most commonly used unsupervised clustering method. Hierarchical clustering was performed using the R package ‘pheatmap’ (version 1.0.12). ROC curves, and the area under the curve (AUC) values were used to illustrate the predictive power.

## Supplementary Information

**Additional file 1: Table S1.** List of tissue-specific plasma-derived 5hmC-modified lncRNA genes.

**Additional file 2: Table S2.** List of 140 tissue-shared 5hmC-modified lncRNA genes.

## Data Availability

The datasets used and analyzed during the present study are available from the corresponding author on reasonable request.

## Abbreviations

5hmC: 5-Hydroxymethylcytosine
AUC: Area under the curve
CART: Classification and regression tree
CC: Colon cancer
CHB: Chronic hepatitis B virus infection
CI: Confidence interval
cfDNA: Cell-free DNA
EC: Esophageal cancer
GC: Gastric cancer
LC: Liver cirrhosis
LncRNAs: Long non-coding RNAs
NSCLC: Non-small-cell lung cancer
RFE: Recursive feature elimination
HCC: Hepatocellular carcinoma
